# The American Psychological Journal

**Published:** 1853-05

**Authors:** 


*The American Psychological Journal, devoted chiefly to the elucidation of Mental Pathology and the Medical Jurisprudence of Insanity.* Conducted by Edward Mead, M. D., Physician to the Cincinnati Retreat for the Insane, etc., etc.

This is the title of a new periodical, the first No. of which has lately appeared, to be issued at Cincinnati, Ohio. It is a bi-monthly publication, of thirty-two pages, the subscription price being but one dollar *per annum.* The editor, Dr. Mead, is a working man, and an excellent writer. The work will be worth more than the subscription price to all who feel an interest in the subjects to which it will be devoted.

